# Reaching is Better When You Get What You Want: Realtime Feedback of Intended Reaching Trajectory Despite an Unstable Environment

**DOI:** 10.3389/fnbeh.2015.00365

**Published:** 2016-01-12

**Authors:** Justin Horowitz, Tejas Madhavan, Christine Massie, James Patton

**Affiliations:** ^1^Neuro-Machine Interaction Laboratory, Bionengineering, University of Illinois at ChicagoChicago, IL, USA; ^2^Robotics Lab, Rehabilitation Institute of Chicago, Sensory Motor Performance ProgramChicago, IL, USA

**Keywords:** movement control, reaching, planning, feedback, intent, intention, desire

## Abstract

Improvements in human-machine interaction may help overcome the unstable and uncertain environments that cause problems in everyday living. Here we experimentally evaluated intent feedback (IF), which estimates and displays the human operator's underlying intended trajectory in real-time. IF is a filter that combines a model of the arm with position and force data to determine the intended position. Subjects performed targeted reaching motions while seeing either their actual hand position or their estimated intent as a cursor while they experienced white noise forces rendered by a robotic handle. We found significantly better reaching performance during force exposure using the estimated intent. Additionally, in a second set of subjects with a reduced modeled stiffness, IF reduced estimated arm stiffness to about half that without IF, indicating a more relaxed state of operation. While visual distortions typically degrade performance and require an adaptation period to overcome, this particular distortion immediately enhanced performance. In the future, this method could provide novel insights into the nature of control. IF might also be applied in driving and piloting applications to best follow a person's desire in unpredictable or turbulent conditions.

## 1. Introduction

Humans often interact with machines in uncertain and complicated environments, such as crowds and traffic, where they must contend with turbulence, moving obstacles, distractions, and disturbances. Despite our capacity to learn and adapt, some environments evolve too quickly or with too much uncertainty for meaningful learning. Human and animal nervous systems intelligently solve many problems by planning ahead (Belen'kii et al., 1967) and suppressing suboptimal actions (Mirabella, 2014), yet in the face of uncertainties we often cannot adequately prevent errors. There is the possibility, however, to exploit additional information from instruments—particularly fast and accurate force sensors—that can measure human machine interactions. Combining sensors with filtering techniques makes it possible to determine a person's underlying *intent*, operationally defined as the motion they would have made had they not been disturbed. While other components of a movement, such as its goal, are also intended (Mirabella, 2014), our work here addresses only the intended trajectory. This intent provides new ways to understand the nature of control and provide novel feedback.

Recent work in our laboratory has attempted to outline a methodology for obtaining estimates of intent (Horowitz and Patton, 2015). This method assumes a model of the dynamics and control of the human arm. Following manipulations of the equations of motion, the method integrates to find a unique estimate of intent. The algorithm recovers the trajectory a person intended to take, even if they were forced away from it due to environmental disturbances. This analysis has enabled us to show how some subjects sometimes alter their intent following exposure to unexpected force pulses.

A new question that arises is whether seeing one's own intent, rather than what actually happens, may be useful. The intent extraction method can be streamlined to allow for real-time estimations of intent that can be presented to the subject as a cursor. Estimated intention may outperform the movement accuracy in the presence of unexpected disturbances. If so, such a method holds great promise in any situation where humans and machines interact as it enables the machine to give the human operator what they want. This human-machine collaboration could outperform what a person can do alone.

Displaying anything other than what truthfully happens is a distortion and a deceit. Like many other visual distortion experiments (Miles and Eighmy, 1980; Pine et al., 1996), intent feedback (IF) introduces a visuomotor discrepancy that may be confusing to the nervous system. Preplanning a specific route may not be necessary, and instead the system might continuously react to any environmental disturbances until it reaches the goal. If people try to achieve a goal while minimizing some measure of cost, it is possible to compute a set of rules for reacting to the environment (Todorov and Jordan, 2002). No specific intended route is needed when using optimal feedback control. While this modeling strategy has been very successful at explaining data, it fundamentally assumes that corrective actions will be taken in response to relevant disturbances. Goals can also be reached at minimal *expected* cost by constructing—and possibly updating—a specific intended route. If no particular trajectory is intended, the nervous system could be unable to recognize IF.

While performance is the best indicator of IF's worth, changes in arm stiffness can provide supporting evidence that subjects are actually getting what they want. Arm stiffness is known to increase during exposure to instability (Franklin et al., 2003) and uncertainty (Takahashi et al., 2001). We anticipated that these changes might also be modulated by the presence of IF. Reducing the effects of environmental instability and presenting the subject with a signal already known to them should relax their arm and make it more compliant. We hypothesized that any elevated arm stiffness from noisy disturbances would decrease while subjects received IF.

In this paper, we describe our streamlined method for real-time IF to which we exposed subjects in an unstable and unpredictable environment. Their ability to perform goal-directed reaching using visual feedback of their hand position was compared against IF. We hypothesized that IF should lead to better performance in the presence of force-based disturbances. Accordingly, we hypothesized that during random disturbances, the intent trajectory should deviate less than the hand trajectory.

## 2. Materials and methods

### 2.1. Intent extraction

The well-known motion control structure of Shadmehr and Mussa-Ivaldi (1994) relates arm trajectory, *q*, to desired arm trajectory, *q*_*d*_, and any external disturbance, *E* using physical parameters of the arm. To show how this model can be algebraically inverted to instead describe desired arm trajectory as a function of arm trajectory and external disturbance, we write it as a torque balance:

(1) $$\begin{array}{l} \underset{\text{Plant}}{\underset{︸}{\overset{\text{Inertia}}{\overset{︷}{M\left(q\right)\ddot{q}}}+\overset{\text{Coriolis, Centripal}}{\overset{︷}{G\left(q,\dot{q}\right)}}}}+E=\underset{\text{Controller}}{\underset{︸}{\overset{\text{Feedforward}}{\overset{︷}{\tau_{ff}}}+\overset{\text{Feedback}}{\overset{︷}{\tau_{fb}}}}} \end{array}$$

Where *M* is the mass matrix, *q* is the joint angles, $\dot{q}$ is joint angular velocities, $\ddot{q}$ is joint angular accelerations, and *G* contains both Coriolis and centripetal effects. Typical applications solve this torque balance for $\ddot{q}$ and use a numerical differential equation solver to predict arm trajectory in the context of a disturbance of interest, a feedback model, and feedforward torques determined by inverse dynamics. Rather than test hypotheses regarding the learning, production, or composition of this feedforward torque, we instead solved for it:

(2) $$\begin{array}{l} \tau_{ff}=M\left(q\right)\ddot{q}+G\left(q,\dot{q}\right)+\;E-\tau_{fb} \end{array}$$

Then we noted that feedforward torque can have a one-to-one correspondence with desired acceleration, ${\ddot{q}}_{d}$:

(3) $$\begin{array}{l} \hat{M}\left(q_{d}\right){\ddot{q}}_{d}+Ĝ\left(q_{d},{\dot{q}}_{d}\right)+Ê=\tau_{ff} \end{array}$$

Hats (ˆ) denote the nervous system's best estimate of a physical quantity. Combining these expressions, suppressing state dependencies, and solving for ${\ddot{q}}_{d}$:

(4) $$\begin{array}{l} {\ddot{q}}_{d}={\hat{M}}^{-1}\left\{M\ddot{q}+G-Ĝ+E-Ê-\tau_{fb}\right\} \end{array}$$

In this form, a differential equation solver can determine *q*_*d*_ as it evolves in time if a few assumptions are made and conditions are met. First, Ê must be modeled or assumed, so we chose Ê = 0. In the presence of a zero mean white noise force disturbance, its mean should be zero, but it is unlikely to be exactly zero and may reflect an average of only the last few exposures (Scheidt et al., 2001). Next, the matrix $\hat{M}\left(q_{d}\right)$ must be invertible, but we ensured this through our choice of workspace. Finally, feedback torque requires a model of arm impedance, which is known to be task-dependent (Gomi and Osu, 1998) and may vary over the course of a reach (Niu et al., 2010). With no prior knowledge of arm impedance for this task-disturbance combination, we presumed the feedback torque model of Shadmehr and Mussa-Ivaldi (1994) anticipating that it is sufficiently accurate or easy to learn (Experiment 1). The experiment was repeated with a lower stiffness estimate (Experiment 2) to explore any dependence on this assumption.

### 2.2. Apparatus

**Figure 1:**
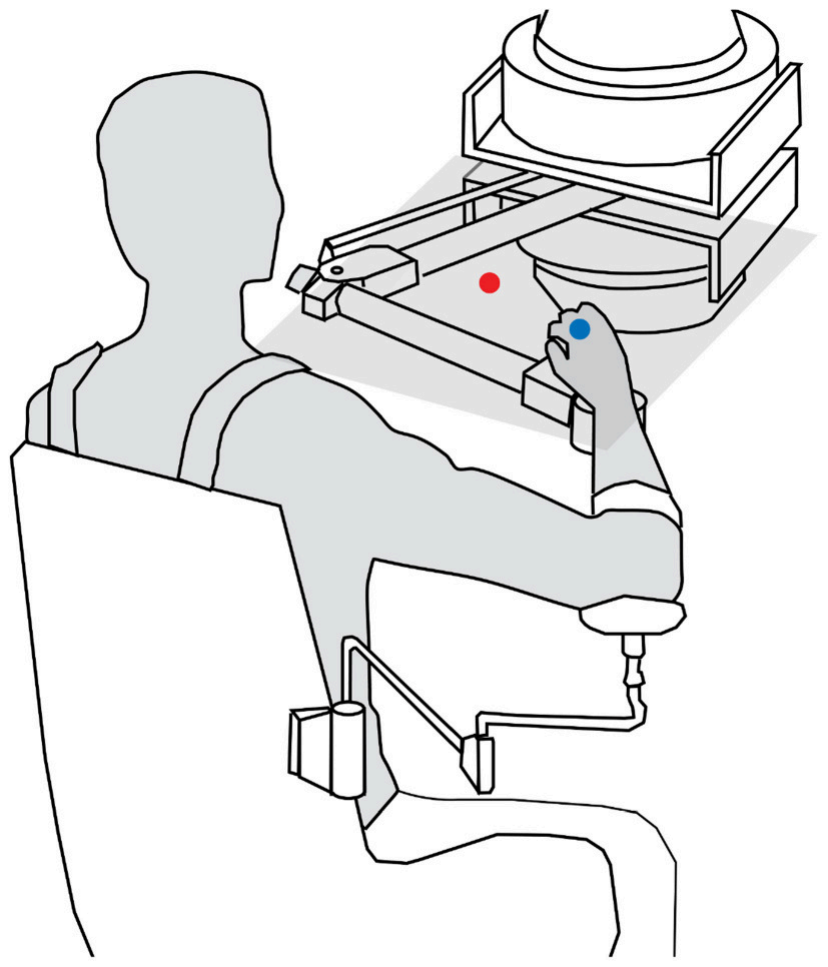
**Subjects were seated at a planar manipulandum capable of measuring position and force as well as rendering forces**. The subject's hand was positioned below an opaque screen so the subject could not see their hand as they reached toward the targets. On the screen, a red circle (the target to reach to) appeared on the screen and subjects were shown a blue circle that either represented the actual position of their hand or their estimated intent as they moved toward the target depending on the movement block.

A planar manipulandum (described in Patton and Mussa-Ivaldi, 2004 and depicted in Figure 1) was programmed to minimize any friction or mass. The MATLAB XPC-TARGET package (MATLAB, 2008) was used to render this force environment at 1000 Hz and data was collected at 1000 Hz. Visual feedback of cursor position was performed at 60 Hz using OpenGL. Closed-loop data transmission time (position measurement to completed rendering to recognition of rendering by the position measurement system) was less than 8 ms, ensuring a visual delay less than one 60 Hz frame. Numerical simulation was performed in real-time using the GNU Scientific Library's odeiv2 driver with Runge-Kutta-Fehlberg (4,5) stepping (Gough, 2009). Visual feedback was given using an opaque screen that prevented subjects from seeing their arm during movement.

### 2.3. Human subjects

The human data trajectories analyzed here are drawn from sixteen subjects who gave informed consent in accordance with Northwestern University Institutional Review Board, which specifically approved this study and followed the principles expressed in the Declaration of Helsinki. Fourteen male and two female right-handed subjects (ages 21–30) performed the reaches with their right arm and were not compensated. Subjects' arm segment lengths were directly measured *in situ* while body mass and handedness were self-reported.

### 2.4. Experimental design

Subjects performed center-out reaches of the right arm to one of three visually-presented targets 15 cm from the center and chosen at 120° intervals. These targets were represented as a red circle with a radius of 1 cm. Target selection was carried out pseudorandomly such that each outer target was visited 16 times in all five blocks of 96 reaches each. During blocks 2 through 4, subjects experienced filtered white noise forces drawn from a white noise generator at 1000 Hz with flat power spectral density of 1 Newton. Forces were then passed through a 4th order low-pass Butterworth filter with cutoff 10π radians per second. In all blocks, except block 3, cursor position (represented as a blue circle with a radius of 1 cm) indicated the subjects' actual hand positions. In block 3, the cursor position indicated the subjects' estimated intents. Once the cursor (blue circle) made contact with the target (red circle), a new target was immediately presented. Both actual hand position and intent were recorded at all times, even though at any given moment only one was visible to the subject.

### 2.5. Dynamic simulation of arm and intended trajectories

Anatomical landmarks and values from Dempster and Center (1955) and Winter (2009) were used to estimate relationships between body mass, limb mass, limb length, limb center of mass, and moment of inertia. Viscosity parameters, *K*_*d*_, were taken from Shadmehr and Mussa-Ivaldi (1994). Stiffness parameters were either taken from Shadmehr and Mussa-Ivaldi (1994) (*K*_*P*1_, Experiment 1) or estimated (*K*_*P*2_, Experiment 2). Expressed in Newton-meters per radian:

(5) $$K_{P1}=\left[\begin{array}{cc} 15 & 6\\ 6 & 16 \end{array}\right]\;K_{P2}=\left[\begin{array}{cc} 8 & 2\\ 2 & 5 \end{array}\right]$$

To estimate this reduced stiffness, a pilot subject was asked to intend to remain still on the center target, *q*_*d*_, while co-contracting as little as possible. *K*_*P*2_ was then calculated from 1 min of white noise forces, *E*, and joint angle traces, *q* as the least squares solution to the system:

(6) $$\begin{array}{l} K_{P2}q_{d}\left(t\right)-q\left(t\right)=Mq\left(t\right)\ddot{q}\left(t\right)+G\left(q\left(t\right),\dot{q}\left(t\right)\right)+E\left(t\right)+K_{D}\dot{q}\left(t\right) \end{array}$$

where *K*_*P*2_ is a 2-by-2 matrix while the state difference and torque are 2-by-60,000 matrices.

Feedback torque was calculated as the sum of viscous and elastic impedances

(7) $$\begin{array}{l} \tau_{fb}=K_{D}\left({\dot{q}}_{d}-\dot{q}\right)-K_{P}\left(q_{d}-q\right) \end{array}$$

with *K*_*P*_ chosen as described and *K*_*D*_ taken from Shadmehr and Mussa-Ivaldi (1994) as expressed below in Newton-meters per radian-second.

(8) $$K_{D}=\left[\begin{array}{cc} 2.3 & 0.09\\ 0.09 & 2.4 \end{array}\right]$$

### 2.6. Metrics and statistical analysis

Trajectories and forces were rotated such that movement and force parallel to the line connecting the previous target (the reach origin) and the presented target were along a *progress* axis, while perpendicular movement and force were along an *error* axis. Reach onset was detected as the moment the cursor's distance from the center of the previous target first exceeded 1 cm. Maximum perpendicular error for a trajectory was the largest error magnitude within 250 ms of reach onset. A scalar stiffness, *k*, was calculated for the *error* axis during this same 250 ms time span by linear regression:

(9) $$\begin{array}{l} F_{e}=më+bė+ke+F_{O} \end{array}$$

Force (*F*_*e*_) and state (ë, ė, *e*) were known. Mass (*m*), viscosity (*b*), and stiffness offset (*F*_0_) terms were calculated, but discarded. While joint stiffness is usually described as a matrix, instantaneous endpoint stiffness in only the error direction is a scalar. This *effective stiffness* metric isolated stiffness in the error direction and facilitated statistical comparison between treatments and blocks. The paired *t*-test was used to detect differences in maximum perpendicular error and stiffness between blocks and treatments at the 5% significance level using the MATLAB statistics toolbox package (MATLAB, 2014).

## 3. Results

**Figure 2:**
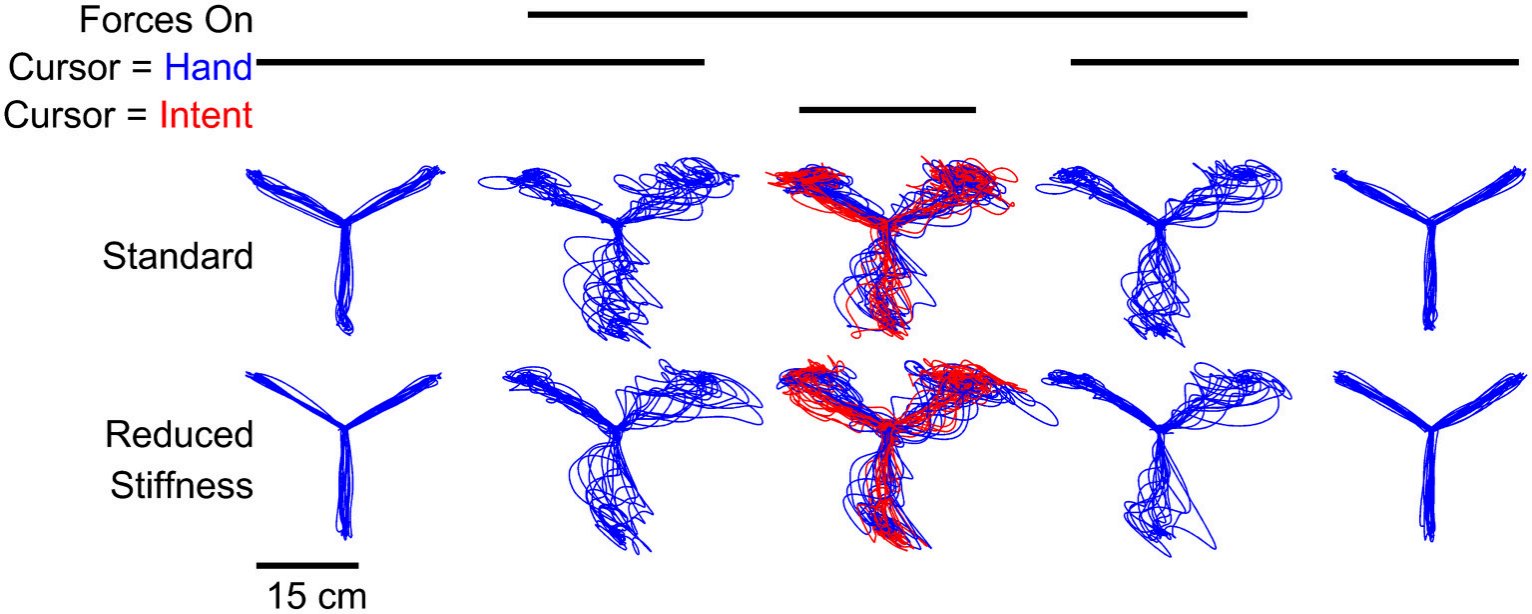
**Typical subjects (one from each experiment) made center-out targeted reaching motions under experimentally varied force and feedback conditions**. Subjects used feedback of either hand motions (blue lines) or estimated intent (red lines) to complete these reaches. Shown also are the measured hand motions in the third block, which were recorded even though they were not visible to the subject (blue) to be compared to intent (red). Intent was estimated using either the standard stiffness model of Shadmehr and Mussa-Ivaldi (1994) or a reduced stiffness model to explore any dependence of reaching stiffness or accuracy on this assumption. The white noise force disturbance was designed to be unpredictable in order to minimize any effect of learning.

**Figure 3:**
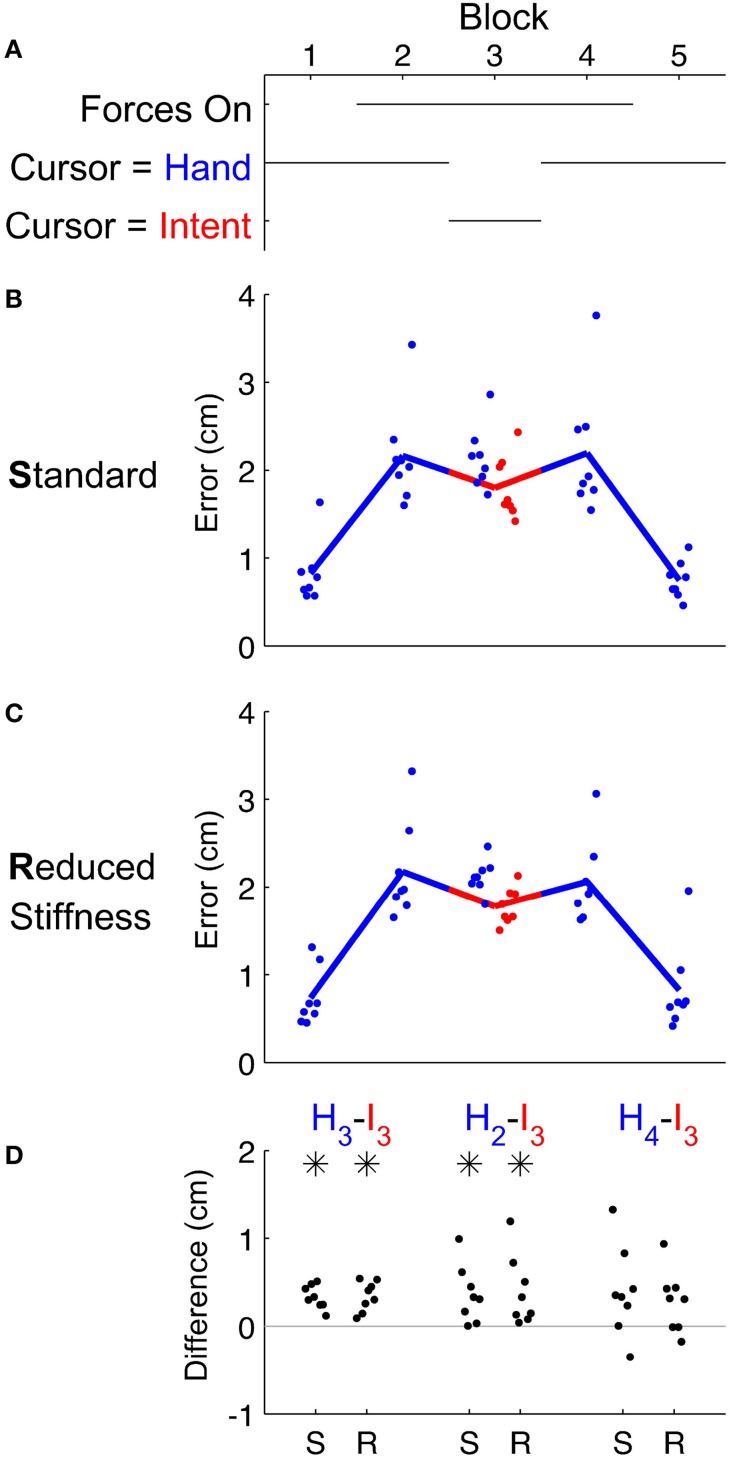
**Subjects' reaching accuracy depended on the presence of force disturbance and the contents of visual feedback (A)**. **(B,C)** Maximum deviation from straight-line reaching calculated during the first 250 ms after the onset of movement revealed that turbulent force disturbance degraded reaching performance. Comparison across feedback modalities revealed that IF (red) in block 3 alleviated performance error relative to hand performance (blue) in blocks 2 and 4. Note that in block 3 we show blue dots indicating the hand's performance, although it was not visible to the subject. **(D)** Several comparisons showing pairwise performance differences amongst blocks 2, 3, and 4. Comparisons between hand performance in block N and IF performance in block 3 are abbreviated as *H*_*N*_−*I*_3_ (asterisks denote *t*-test significance at α = 0.05 level). Performance did not appear to depend on the choice between the standard stiffness model of Shadmehr and Mussa-Ivaldi (1994) (**B**, and “S” labels in **D**) or a reduced stiffness (**C**, and “R” labels in **D**) to determine intent.

As expected, the model was able to deduce an intended trajectory and all subjects were able to use this estimate of their intent to perform targeted reaching while experiencing turbulent forces (Figure 2). There were no obvious changes in performance using intent over time. We also observe no after-effects of either IF or the forces. In block 3 where IF replaced visual feedback of the hand, the intent significantly (*p* = 0.02) outperformed the hand itself. For this intent estimate to be useful, subjects should perform better when using IF than when using feedback of the hand. Comparing the hand's performance in block 2 (Figure 3B) to IF's performance in block 3, subjects performed significantly (*p* = 0.02) better using their estimated intent. While subjects also performed better using IF in block 3 than using their hand in block 4, this difference was not significant (*p* = 0.07).

**Figure 4:**
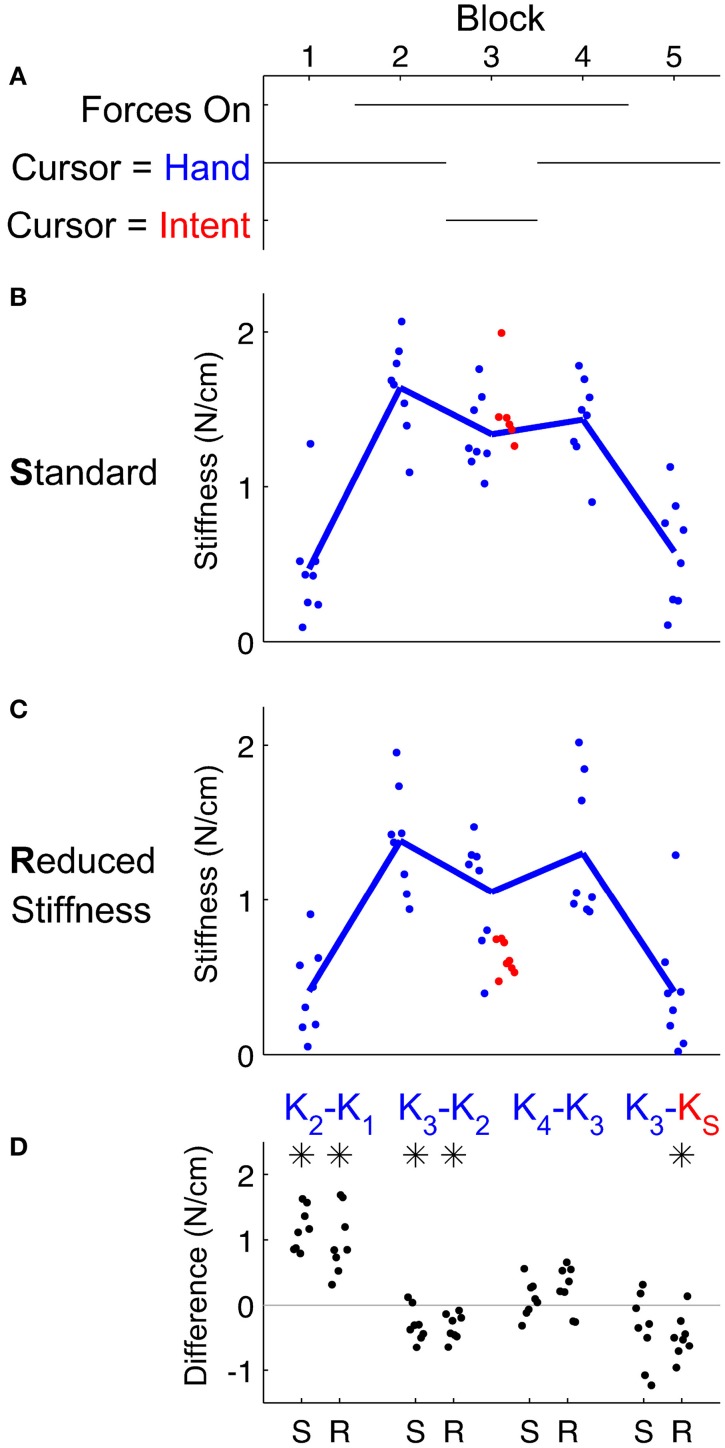
**Subjects' effective stiffness depended on the presence of force disturbance and the contents of visual feedback (A)**. **(B,C)** Effective stiffness, K, calculated by linear regression during the first 250 ms after the onset of movement revealed that turbulent force disturbance increased this stiffness. **(D)** Comparisons between treatment conditions revealed that exposure to turbulent forces caused significant stiffening, but IF could significantly alleviate arm stiffness. As in Figure 3, the estimated arm stiffness in block 3 significantly depended on our choice of either the classic stiffness model of Shadmehr and Mussa-Ivaldi (1994) (**B**, “S” labels) or a reduced stiffness (**C**, “R” labels) to determine intent. Significant differences were determined by paired *t*-test at the α = 0.05 significance level and are denoted by an asterisk.

Interestingly, our measure of effective stiffness changed dramatically across the experimental conditions. Subjects stiffened significantly (*p* < 0.001) in response to white noise forces (compare stiffness in blocks 1 and 2 of Figure 4B). Next, subjects' effective stiffness significantly (*p* = 0.01) decreased when IF replaced the hand location as their cursor (compare blocks 2 and 3 in Figure 4). This decrease did not return stiffness to undisturbed levels, and it remained even after feedback of the hand resumed (comparing blocks 3 and 4). Subjects appear to have adjusted their stiffness in response to the stiffness used by IF. Subject's stiffness's did not significantly (*p* = 0.1) differ from the stiffness of the standard model when it was used by IF. Finally, subjects reported that this IF treatment of the cursor feedback made it easier and they felt more relaxed. Detailed statistics of these differences are summarized in Table 1.

**Table 1:** **Error and stiffness change with feedback type and presence of disturbance**.

	***T*(7)**	***P***	**Mean**	**SEM**
**ERROR COMPARISONS, EXPERIMENT 1: STANDARD STIFFNESS**				
Hand (Block 3)—Intent (Block 3)	7.11	<0.01	0.33 cm	0.05 cm
Hand (Block 2)—Intent (Block 3)	3.00	0.02	0.36 cm	0.13 cm
Hand (Block 4)—Intent (Block 3)	2.15	0.07	0.40 cm	0.15 cm
**ERROR COMPARISONS, EXPERIMENT 2: REDUCED STIFFNESS**				
Hand (Block 3)—Intent (Block 3)	5.69	<0.01	0.34 cm	0.06 cm
Hand (Block 2)—Intent (Block 3)	2.80	0.03	0.40 cm	0.15 cm
Hand (Block 4)—Intent (Block 3)	2.26	0.06	0.28 cm	0.13 cm
**STIFFNESS COMPARISONS, EXPERIMENT 1: STANDARD STIFFNESS**				
Hand Stiffness (Block 2)—Hand Stiffness (Block 1)	10.2	<0.01	1.17 N/cm	0.12 N/cm
Hand Stiffness (Block 3)—Hand Stiffness (Block 2)	–3.24	0.01	–0.30 N/cm	0.10 N/cm
Hand Stiffness (Block 4)—Hand Stiffness (Block 3)	0.98	0.36	0.09 N/cm	0.10 N/cm
Hand Stiffness (Block 3)—Model Stiffness (*K*_*P*1_)	–1.92	0.10	–0.37 N/cm	0.21 N/cm
**STIFFNESS COMPARISONS, EXPERIMENT 2: REDUCED STIFFNESS**				
Hand Stiffness (Block 2)—Hand Stiffness (Block 1)	5.53	<0.01	0.97 N/cm	0.19 N/cm
Hand Stiffness (Block 3)—Hand Stiffness (Block 2)	–4.75	<0.01	0.33 N/cm	0.07 N/cm
Hand Stiffness (Block 4)—Hand Stiffness (Block 3)	2.03	0.08	0.25 N/cm	0.13 N/cm
Hand Stiffness (Block 3)—Model Stiffness (*K*_*P*2_)	3.69	<0.01	0.43 N/cm	0.12 N/cm

As we found evidence of IF dramatically reducing effective arm stiffness, we next examined whether we could reduce effective arm stiffness even more. We asked a second group of subjects to perform the same experiment, except that the arm model used by IF was adjusted to assume a greatly reduced stiffness of the arm. Surprisingly, we found the same performance benefits (Figure 3C), but effective stiffness reduced even more (Figure 4C). Performance using IF in block 3 again significantly outperformed the hand in block 3 (*p* < 0.001) and the hand in block 2 (*p* = 0.02). We found the same beneficial effect relative to block 4, but this difference was not significant (*p* = 0.06). Moreover, this reduced stiffness IF allowed subjects to relax to approximately two-thirds the effective stiffness of Shadmehr and Mussa-Ivaldi (1994) during block 3 (*p* = 0.004). As before, stiffness increased following exposure to white noise forces (*p* < 0.001) that was significantly (*p* = 0.01) alleviated by IF. Removal of IF was still associated with an increase in arm stiffness, but this increase was not significant (*p* = 0.08). Subjects who received this lower stiffness IF also reported that IF was easier and allowed them to greatly relax. In other words, subjects reduced arm stiffness to accommodate the model.

## 4. Discussion

The work presented here highlights the use of a novel visual distortion of the cursor that leads to superior performance in a hand-eye coordination task in the presence of random disturbances. This real-time distortion marks the estimated intent of the subject rather than the hand location in order to make movements easier. Although exposure to random forces hindered subjects reaching accuracy and increased their arm stiffness, replacing the veridical feedback with intent feedback (IF) improved accuracy and decreased stiffness. While other visual distortions typically degrade performance and require an adaptation period to overcome, IF immediately enhanced performance. This type of feedback may be a new method for enhancing performance in human-machine interactions, and also sheds light on how the nervous system uses visual feedback.

The most striking result is that although the nervous system sees an untruth about where the end-effector is, it appears to be effective for improving performance. The IF presentation is one of many visuomotor discrepancies in hand-eye coordination tasks, yet this one does not degrade performance and does not require adaptation. Not all perceptual lies appear to be unwanted. Two possibilities explain this result: either the central nervous system was able to adapt to this new feedback within a single reach, or the means to make use of this signal were already available. For instance, IF may mimic efference copy. The performance variability inherent in white noise force disturbances complicated our observations of the learning process, but simple examination showed there were no obvious differences in performance between the first and final exposures to IF. There were also no obvious after-effects from exposure to IF (Figure 1, final panels). The simplest explanation for this is that IF approximates a signal already known to the brain: the path planned for the hand. In addition to its promise in performance enhancement, IF represents a novel means of revealing and studying the mechanisms of motor planning and motor control.

While IF alleviated the increased stiffness caused by exposure to random forces, stiffness remained significantly above baseline levels. Many explanations are reasonable. In particular, we hypothesized that subjects would adapt their own arm stiffness to decrease conflict with the stiffness model used to estimate their intent and thereby increase the accuracy of the estimate, and while the data did support this conclusion the effect was not strong. Alternatively, inaccuracy and incompleteness of the simple models used might have resulted in an less accurate estimate of intent. Since noise and performance inaccuracy can both lead to co-contraction, this may account for the residual stiffness. Finally, as subjects were not cued regarding the onset or removal of IF, the residual co-contraction may have been a precaution against the resumption of veridical feedback.

The ease with which subjects could make use of their estimated intent provides strong preliminary evidence that a specific intended trajectory was computed for the hand even when reaching in a highly variable environment. While recent work has identified kinematic constraints unnecessary for a task (Mistry et al., 2013), this is the first direct evidence that the entire trajectory is controlled even in the absence of specific instructions or constraints. While portions of the intended trajectory are surely computed before the onset of movement, movement intent is not finalized before the onset of movement and is not strictly ballistic. There is mounting evidence of multiple corrective actions formulated after movement onset in recent literature. For example, Mirabella (2008) compared onset and movement times during a task that could be countermanded, and found that quicker onset times were compensated by longer movement times, most likely due to the need for on-line proactive adjustments in anticipation of known task demands. Such a context effect has also been recently replicated on Parkinsons patients (Mirabella et al., 2013).

It is important to distinguish the signal we are exploring from others that may use the word “intent.” One source of complexity is that formation of actions is a multi-step process in which several brain regions contribute. The intent we refer to here is not the goal-oriented intent that might temporally precede the computation of the motor plan (Haggard, 2008; Mirabella, 2014). The intended trajectory and goal remain malleable and can change (or be suppressed) even after execution (Ghez et al., 1997). Work by Mirabella et al. (2013) showed that inhibition is important for quickly aborting, interrupting, and re-planning motion after its onset. In contrast, our version of intent focuses on the final stage of planning and hence the command at the present-time. This is after the nervous system has completed any further checks and released the plan. In a sense, the intent we provide is in the present and not pre-planned (for the future) or post-processed *a posteriori* (in the past).

It is also important to distinguish this from other methods that attempt to determine the ultimate target of action (the goal). A number of human-computer approaches strive to identify, for example, the final target of a movement (Ziebart et al., 2012). In our rather limited task with only three possible reaching targets, identification was trivial. We analyzed this on our task and were able to easily identify the target with 95% visuomotor efficiency (closely related to accuracy, Sakitt, 1980) within the first 80 ms of motion, regardless of whether we used the hand or IF. In contrast to these target-prediction methods, our approach allowed for the *instantaneous* determination of the intended hand location. The novelty of such instantaneous detection created the prospect of real-time feedback in human-machine interactions.

Intent Feedback might facilitate human-machine collaboration and artificial performance augmentation by enabling the machine to preserve an operator's intent while canceling unexpected disturbances from the environment. This should reduce the demand on the human operator and increase performance—especially in environments with rapidly changing conditions. This assistance goes beyond environment cancellation by also accounting for any errors the operator might make based on their expectation of disturbance.

While IF holds promise, it also has strong limitations. IF is entirely dependent on the accuracy of the models used. While we were able to leverage measurements of cadaver anthropometrics, average tendencies do not capture individual variability. Similarly, the model of Shadmehr and Mussa-Ivaldi (1994) appears to have accurately captured the mean tendencies (Figure 3D, rightmost comparison) without accounting for variation among individuals or variation over time. Techniques that could estimate changing stiffness, perhaps even in real-time, would greatly increase the accuracy and utility of IF. While IF is limited by the accuracy of the models used, many candidate models are available and may outperform the simple model we investigated here in this preliminary study.

More broadly, this IF approach may be useful in any situation where some model of the dynamics is available and disturbances can be measured. For example, brain-computer interfaces may need to address measurable common-mode electrical artifacts, such as the electromagnetic disturbances that occur from lights being turned on. In cases where a disturbance can be measured due to its similar effect across all sensors, IF allows the interface to respond in a manner congruent with the user's intent. This is especially important when simply canceling the disturbance is not sufficient or practical, such as an exoskeleton's user intending to crouch down during an earthquake rather than trying to remain upright.

In any case, intent feedback led to performance benefits for subjects moving in a changing, uncertain environment. In addition to increasing subjects' accuracy, IF may have allowed subjects to reach their goals with less effort as arm stiffness decreased. IF provides a novel form of feedback that may facilitate new insights into the nature of motor control and allows a machine to collaborate more effectively with a human user.

## Author contributions

All authors designed the work, analyzed the data, drafted and revised the manuscript, approved the final version, and agree to be held accountable for all aspects. TM and CM collected the data.

## Funding

Funded By NIH R01-NS053606 and NIDILRR 90RE5010-01.

### Conflict of interest statement

The authors declare that the research was conducted in the absence of any commercial or financial relationships that could be construed as a potential conflict of interest.
